# Does special education in palliative medicine make a difference in end-of-life decision-making?

**DOI:** 10.1186/s12904-018-0349-6

**Published:** 2018-07-18

**Authors:** Reetta P. Piili, Juho T. Lehto, Tiina Luukkaala, Heikki Hinkka, Pirkko-Liisa I. Kellokumpu-Lehtinen

**Affiliations:** 1 Faculty of Medicine and Life Sciences, University of Tampere, Tampere, Finland; 2 Department of Oncology, Tampere University Hospital, Tampere, Finland; 3 Department of Oncology, Tampere University Hospital, Palliative Care Unit, Teiskontie 35, R-building, 33520 Tampere, Finland; 4 Research and Innovation Center, Tampere University Hospital, Tampere, Finland; 5 Health Sciences, Faculty of Social Sciences, University of Tampere, Tampere, Finland; 6 Rehabilitation Center Apila, Kangasala, Finland

**Keywords:** Decision-making, Terminal care, Education, Palliative medicine, Life support care

## Abstract

**Background:**

Characteristics of the physician influence the essential decision-making in end-of-life care. However, the effect of special education in palliative medicine on different aspects of decision-making in end-of-life care remains unknown. The aim of this study was to explore the decision-making in end-of-life care among physicians with or without special competency in palliative medicine (cPM).

**Methods:**

A questionnaire including an advanced lung cancer patient-scenario with multiple decision options in end-of-life care situation was sent to 1327 Finnish physicians. Decisions to withdraw or withhold ten life-prolonging interventions were asked on a scale from 1 (definitely would not) to 5 (definitely would) – first, without additional information and then after the family’s request for aggressive treatment and the availability of an advance directive. Values from chronological original scenario, family’s appeal and advance directive were clustered by trajectory analysis.

**Results:**

We received 699 (53%) responses. The mean values of the ten answers in the original scenario were 4.1 in physicians with cPM, 3.4 in general practitioners, 3.4 in surgeons, 3.5 in internists and 3.8 in oncologists (*p* < 0.05 for physicians with cPM vs. oncologists and *p* < 0.001 for physicians with cPM vs. others). Younger age and not being an oncologist or not having cPM increased aggressive treatment decisions in multivariable logistic regression analysis. The less aggressive approach of physicians with cPM differed between therapies, being most striking concerning intravenous hydration, nasogastric tube and blood transfusions. The aggressive approach increased by the family’s request (*p* < 0.001) and decreased by an advance directive (*p* < 0.001) in all physicians, regardless of special education in palliative medicine.

**Conclusion:**

Physicians with special education in palliative medicine make less aggressive decisions in end-of-life care. The impact of specialty on decision-making varies among treatment options. Education in end-of-life care decision-making should be mandatory for young physicians and those in specialty training.

**Electronic supplementary material:**

The online version of this article (10.1186/s12904-018-0349-6) contains supplementary material, which is available to authorized users.

## Background

Rapid developments in medicine have allowed many interventions for patients with very advanced diseases. At the same time, the difficulty of choosing worthwhile therapies for each patient has led to the use of non-beneficial treatments among dying patients at their end-of-life (EOL) [1]. In contrast, well-timed palliative care improves patients’ quality of life and symptom control and reduces invasive procedures and costs [2–8].

Appropriate decision-making is mandatory in high-quality EOL-care to prevent non-beneficial treatments and relieve suffering. The decisions include, but are not limited to, statements on cardiopulmonary resuscitation, parenteral fluids, and diagnostic tests. This decision-making is a challenging process involving many ethical, legal, medical and psychological aspects [9–16].

Physicians’ decisions vary concerning different interventions. In a Scandinavian study, 57% of intensive care physicians would continue intravenous hydration, but only 5% of them measured blood glucose during EOL-care [17]. Physicians also decide to withdraw therapies less frequently than to withhold them, probably because they feel withdrawal is more difficult and ethically problematic [18–21].

In addition to medical facts and personal characteristics, education and specialty of the physician influence the complex decision-making in EOL [11]. Although education in palliative care increases the knowledge and skills needed to perform high-quality EOL-care [22–27], the effectiveness of special training in palliative medicine (PM) on different aspects of decision-making in EOL-care remains unknown.

Most patients wish their closest ones to be involved in EOL decision-making, and discussions with the family are essential [28–30]. The families’ opinions are also shown to influence physicians’ decisions [12, 31, 32], although discordance between patients’ wishes, caregivers’ preferences and caregivers’ predictions of patients’ preferences may exist [28, 33]. Advance directives reinforce patients’ participation and help with decision-making [31, 32, 34]. However, there are variations in how advance directives are understood and taken into account [12, 35–37].

The aim of our study was to examine whether special education in PM affects decision-making in EOL-care, as evaluated by a hypothetical patient scenario with different alternatives. The impact of family requests, written advance directives, and physicians’ background factors on their decisions were analysed.

## Methods

### Participants

A postal survey with a questionnaire was provided to 1327 Finnish physicians in autumn 2015. The sample consisted of 500 general health care practitioners (GPs), 300 surgeons, and 300 internists randomly selected from the register of the Finnish Medical Association. The sample size is similar to our previous studies done sixteen years ago and is based on the distribution of different specialities in Finland, which has remained largely unchanged over the years studied [10–12, 38, 39]. In addition, the questionnaire was send to all Finnish oncologists (*n* = 158) and all physicians with a special competency in PM (*n* = 82), excluding those with a mailing proscription (*n* = 23). Two reminders were send to nonrespondents.

A cover letter including an introduction to the study and an assurance of anonymity and voluntariness was mailed together with the questionnaire. This study was approved by the Regional Ethics Committee of Tampere University Hospital, Finland (R15101).

### Special competency in palliative medicine

In Finland, postgraduate training in PM leads to a certification for special competency in PM (cPM) awarded by the Finnish Medical Association [40]. Finnish physicians are allowed to start this postgraduate training after working at least 2 years as a physician. This special training consists of 150 h of theoretical education in different aspects of PM, 200 patient interactions in palliative care, 2 years of clinical practice including a working period in a specialized palliative care unit for a minimum of 3 months, and a final written examination.

### Questionnaire

The questionnaire has been previously used and validated with Finnish physicians. A pilot study was done in January 1999. The questionnaire was sent to 45 physicians (health care practitioners and specialists) twice at two-week intervals in order to test the reliability of the responses to patient scenarios and the questions on attitudes and values. Thirty physicians returned two acceptable questionnaires. The value of kappa coefficient for an acceptable scenarios or questions was determined to be more than 0.40, which is a commonly accepted limit for reliability. [10–12, 38]

The questionnaire includes seven hypothetical patient scenarios together with questions concerning responders’ background, personal features, and attitudes. In this study, we included one of the patient scenarios designed to study doctors’ treatment decisions in the EOL-care. In addition, questions about the responders’ own advance directives, experience in EOL-care among relatives, treatment of EOL patients within 2 years, availability of professional supervision, chief position and financial responsibility at work together with age and sex were used as background factors. The parts of the questionnaire used in this study are available as an Additional file 1.

### Case scenario

The scenario presented a 62-year-old male patient with pulmonary cancer and metastases. He was admitted to hospital ward and received high-dose morphine medication. Due to respiratory weakening, he had become comatose the night before.

He also suffered from severe anaemia and had abundant pleural effusion and fever.

After the presentation of the patient scenario, there was a question about the treatment decision: Which of the following treatments already started (*) or planned would you withhold or withdraw? In the first situation, there was no possibility of discussing the matter with the family and there was no advance directive. The decision responses were expressed on a scale from 1 (I definitely would not) to 5 (I definitely would). The treatments were a) antibiotics (*); b) mechanical ventilation (*); c) blood transfusion; d) pleural drainage; e) chest x-ray examination; f) laboratory tests; g) intravenous hydration (*); h) nasogastric tube (*); i) thrombosis prophylaxis (*); and j) supplementary oxygen (*).

After the original patient scenario, two alternatives with extra information were provided: 1) the patient’s daughters come to you distressed and crying, expressing their hope that everything possible will be done to save their father’s life; 2) there is a written advance directive in the patient’s medical chart in which he expresses his wish that all active treatment should be withdrawn if there is no hope for recovery. After each of these alternatives the same questions (with the same treatment options as in the original scenario) were asked. Questions were asked to be answered in the given order and not to change answers once decided.

### Statistical analysis

**Fig. 1:**
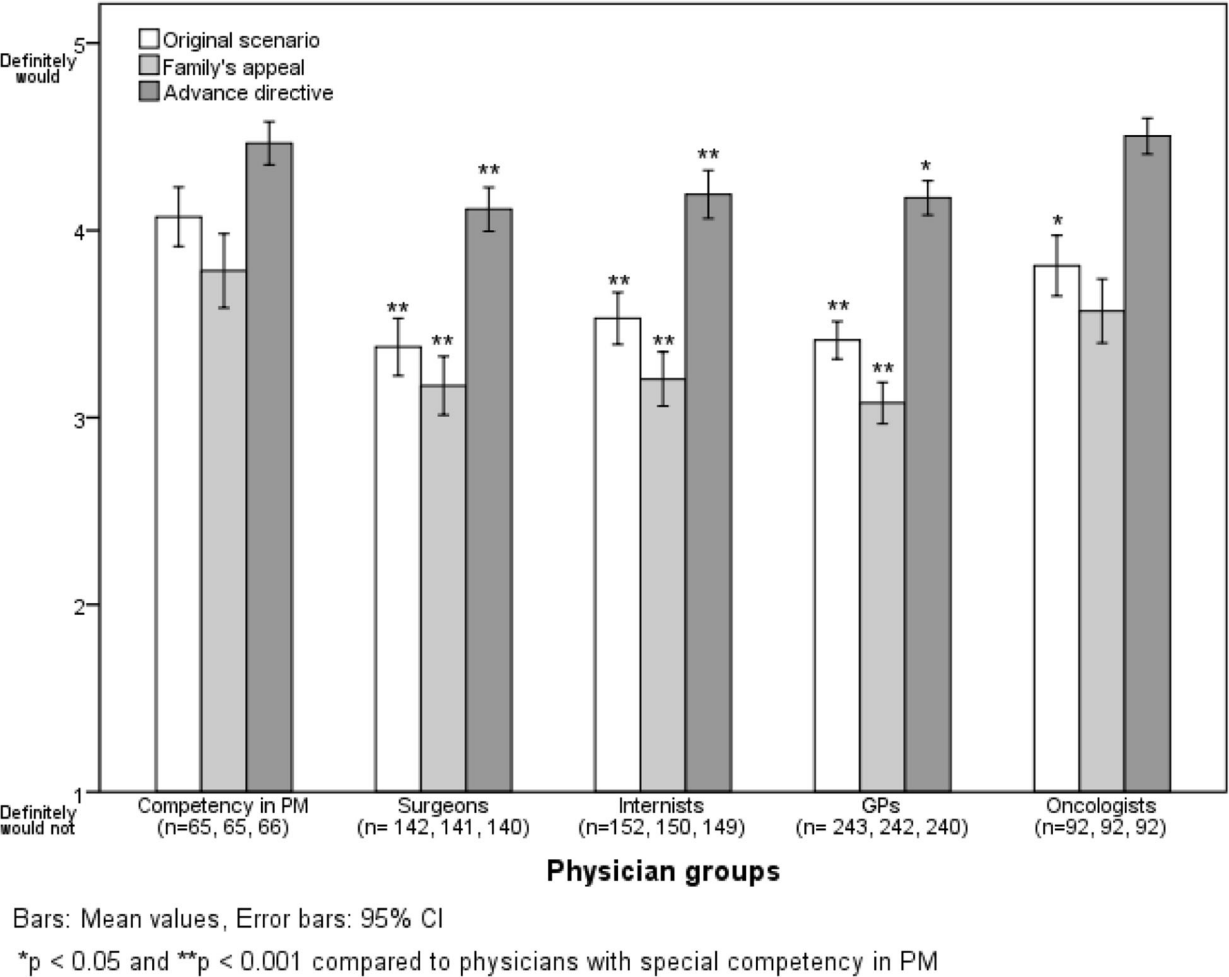
Mean values of all ten answers concerning willingness to withhold or withdraw therapies (scale from 1 = definitely would not to 5 = definitely would) in the patient case according to different scenarios and physician groups

Different responder groups were compared by t-test for normally distributed continuous variables (Fig. 1) and by chi-square or Fisher’s exact tests when appropriate for categorical variables (Tables 2 and 3). The answers on the 5-step Likert scale in the scenarios were converted to a 2-step scale: 1–3, “would not withdraw or withhold and don’t know” and 4–5, “would withdraw or withhold”. Measured mean distributions of the chronological original scenario, family’s appeal and advance directive values were clustered by trajectory analysis [41]. The trajectories were created according to the measurements of mean values in each responder as a continuous outcome measure. The analyses undertaken were latent class mixture models of quadratic trajectories including a random intercept and concomitant variables. Models were fitted by using the flexmix package [42] of the statistical program R, version 3.3.0, from the R Foundation for Statistical Computing (R Development Core Team. R: A language and environment for statistical computing. R Foundation for Statistical Computing, Vienna, Austria, 2008, ISBN 3–900,051–07-0, URL). Relative goodness of fit was assessed using Bayesian information Criteria.

Factors (Table 4) affecting the willingness to continue or start therapies (belonging to trajectory groups 3 or 4) compared to withhold or withdraw therapies (belonging to trajectory groups 1 or 2) were examined by univariate and age-adjusted logistic regression models results shown by odds ratios (OR) with 95% confidence intervals (CI). Additionally, a multivariable model, where variables were added simultaneously into the model, was performed for variables with statistical significance under 0.20 in age-adjusted model. Two-sided *p*-values of less than 0.05 were accepted as statistically significant. Data-analyses were performed using IBM SPSS Statistics for Windows, Version 23.0. Armonk, NY: IBM Corp. Released 2014.

## Results

### Responders

Altogether, 699 valid responses were achieved (response rate 53%). The response rate ranged from 82% among physicians with cPM to 47% among surgeons. Characteristics of the responders are presented in Table 1. A majority of the responders were women, except in the group of surgeons. The median age of the responders was 52 years (interquartile range 43–58), with slight variations between the groups. Most of the GPs worked at out-patient clinics (85%), while the others mainly worked at hospitals (66–87%).

**Table 1:** Characteristics of the participants

	Competency in PM		Surgeons		Internists		GPs		Oncologists		Total	
Number (% of total)	67	(10)	142	(20)	153	(22)	245	(35)	92	(13)	699	(100)
Response rate, %	82		47		51		49		63		53	
Female, n (%)	57	(85)	47	(33)	81	(53)	173	(71)	73	(79)	431	(62)
Median age (IQR)	55	(49–58)	52	(44–59)	53	(46–59)	49	(38–57)	49	(41–56)	52	(43–58)
Age distribution, n (%)												
< 35	0	(0)	4	(3)	4	(3)	42	(17)	2	(2)	52	(7)
35–49	20	(30)	52	(37)	51	(33)	84	(34)	46	(50)	253	(36)
> 49	47	(70)	86	(61)	98	(64)	119	(49)	44	(48)	394	(56)
Years from graduation, median (IQR)^a^	27	(21–32)	26	(17–34)	26	(20–32)	21	(9–31)	22	(14–29)	25	(15–32)

PM, Palliative Medicine, GP, General Practitioner, IQR, Interquartile Range

^a^For nine participants year of graduation was not available

### Overall willingness to withhold or withdraw therapies

The mean values of all ten answers according to the three alternatives in the case scenario are shown in Fig. 1. Physicians with cPM were most willing to withhold and withdraw interventions, especially compared to GPs, internists and surgeons. The family’s appeal significantly increased the willingness to start or continue life-prolonging therapies in all physician groups, whereas the advance directive decreased it (*p* < 0.001 compared to the original scenario).

### Decisions concerning individual treatments

Physicians with cPM were more willing to withdraw and withhold most of the individual interventions, compared to the others (Tables 2 and 3). This difference in decision-making was most striking in withdrawing intravenous hydration, removing nasogastric tube and withholding blood transfusions. In contrast, some decisions (e.g., withdrawing oxygen or antibiotics) varied only slightly between the physicians with cPM and others. Mechanical ventilation was withdrawn by most of the physicians, while supplementary oxygen was frequently continued by all responders.

**Table 2:** Number and proportion (%) of physicians deciding to withdraw a treatment in the patient scenario according to physician groups

Treatment	Scenario	Competency in PM		Surgeons		Internist		GPs		Oncologists		P-value^a^
Antibiotic	Original scenario	41	(65)	72	(51)	78	(51)	128	(53)	45	(54)	0.399
	Family’s appeal	33	(50)	55	(39)	55	(36)	89	(37)*	34	(37)	0.353
	Advance directive	58	(87)	116	(83)	122	(82)	204	(84)	82	(89)	0.641
Mechanical ventilation	Original scenario	63	(96)	119	(85)*	135	(89)	195	(81)*	83	(91)	0.008
	Family’s appeal	59	(92)	113	(80)*	126	(83)	175	(72)*	76	(84)	0.002
	Advance directive	67	(100)	130	(92)*	147	(98)	228	(94)*	90	(99)	0.011
Intravenous hydration	Original scenario	43	(65)	31	(22)**	39	(26)**	85	(35)**	42	(46)*	< 0.001
	Family’s appeal	26	(40)	21	(15)**	19	(13)**	58	(24)*	34	(37)	< 0.001
	Advance directive	58	(88)	64	(46)**	84	(56)**	162	(67)*	75	(82)	< 0.001
Nasogastric tube	Original scenario	62	(95)	85	(60)**	98	(65)**	161	(67)**	63	(69)**	< 0.001
	Family’s appeal	60	(92)	76	(54)**	87	(58)**	126	(53)**	64	(70)*	< 0.001
	Advance directive	64	(97)	105	(75)**	126	(84)*	203	(84)*	82	(89)	0.001
Thrombos prophylaxis	Original scenario	55	(85)	108	(76)	105	(69)*	151	(62)*	73	(80)	< 0.001
	Family’s appeal	52	(80)	104	(74)	95	(64)*	128	(53)**	67	(73)	< 0.001
	Advance directive	63	(96)	121	(88)	127	(85)*	199	(82)*	86	(94)	0.013
Supplementary oxygen	Original scenario	11	(16)	16	(11)	23	(15)	14	(6)*	11	(12)	0.019
	Family’s appeal	12	(18)	14	(10)	13	(9)*	12	(5)	6	(7)*	0.011
	Advance directive	20	(30)	40	(28)	46	(31)	51	(21)	27	(29)	0.189

PM, Palliative Medicine, GP, General Practitioner

^a^Global p-value across all physician groups

*p < 0.05 and **p < 0.001 pair-wise comparison to physicians with special competency in PM

**Table 3:** Number and proportion (%) of physicians deciding to withhold an intervention in the patient scenario according to physician groups

Treatment	Scenario	Competency in PM		Surgeons		Internists		GPs		Oncologists		P-value^a^
Blood transfusion	Original scenario	60	(91)	98	(70)*	108	(72)*	185	(76)*	72	(78)*	0.011
	Family’s appeal	55	(85)	90	(64)*	85	(56)**	153	(64)*	67	(76)	< 0.001
	Advance directive	66	(99)	132	(94)	136	(91)	227	(94)	90	(98)	0.134
Pleural drainage	Original scenario	43	(65)	59	(42)*	85	(56)	99	(41)**	58	(64)	< 0.001
	Family’s appeal	41	(63)	57	(40)*	73	(49)	89	(37)**	51	(56)	< 0.001
	Advance directive	53	(79)	103	(73)	119	(80)	179	(74)	82	(89)	0.030
Chest X-ray	Original scenario	51	(77)	66	(47)**	88	(58)*	119	(60)**	67	(73)	< 0.001
	Family’s appeal	44	(67)	61	(43)*	74	(49)*	29	(38)**	59	(65)	< 0.001
	Advance directive	59	(88)	111	(79)	124	(83)	194	(81)	90	(98)*	0.001
Laboratory tests	Original scenario	49	(74)	70	(59)*	87	(57)*	121	(50)*	59	(64)	0.002
	Family’s appeal	40	(61)	60	(43)*	67	(45)*	85	(35)**	51	(56)	< 0.001
	Advance directive	58	(87)	115	(81)	119	(78)	190	(78)	88	(96)*	0.006

PM, Palliative Medicine, GP, General Practitioner

^a^Global p-value across all physician groups

*p < 0.05 and **p < 0.001 pair-wise comparison to physicians with special competency in PM

The daughters’ request for “everything to be done” (the family’s appeal) increased the willingness to continue or start each life-prolonging treatment, with the only exception the use of oxygen among cPMs. The daughters’ request had the largest influence on the decisions concerning intravenous hydration and diagnostic tests (Table 2).

The availability of the advance directive markedly moved decisions towards withdrawing and withholding treatments. Although the differences between responder groups diminished, the physicians with cPM and the oncologists still had the least aggressive approach. Nearly all physicians withdrew mechanical ventilation, discontinued thrombosis prophylaxis and withheld blood transfusion. However, over one third of the physicians without cPM continued intravenous hydration, and supplementary oxygen was frequently continued by all physicians.

### Trajectory analysis and factors associated with aggressive treatment decisions

**Fig. 2:**
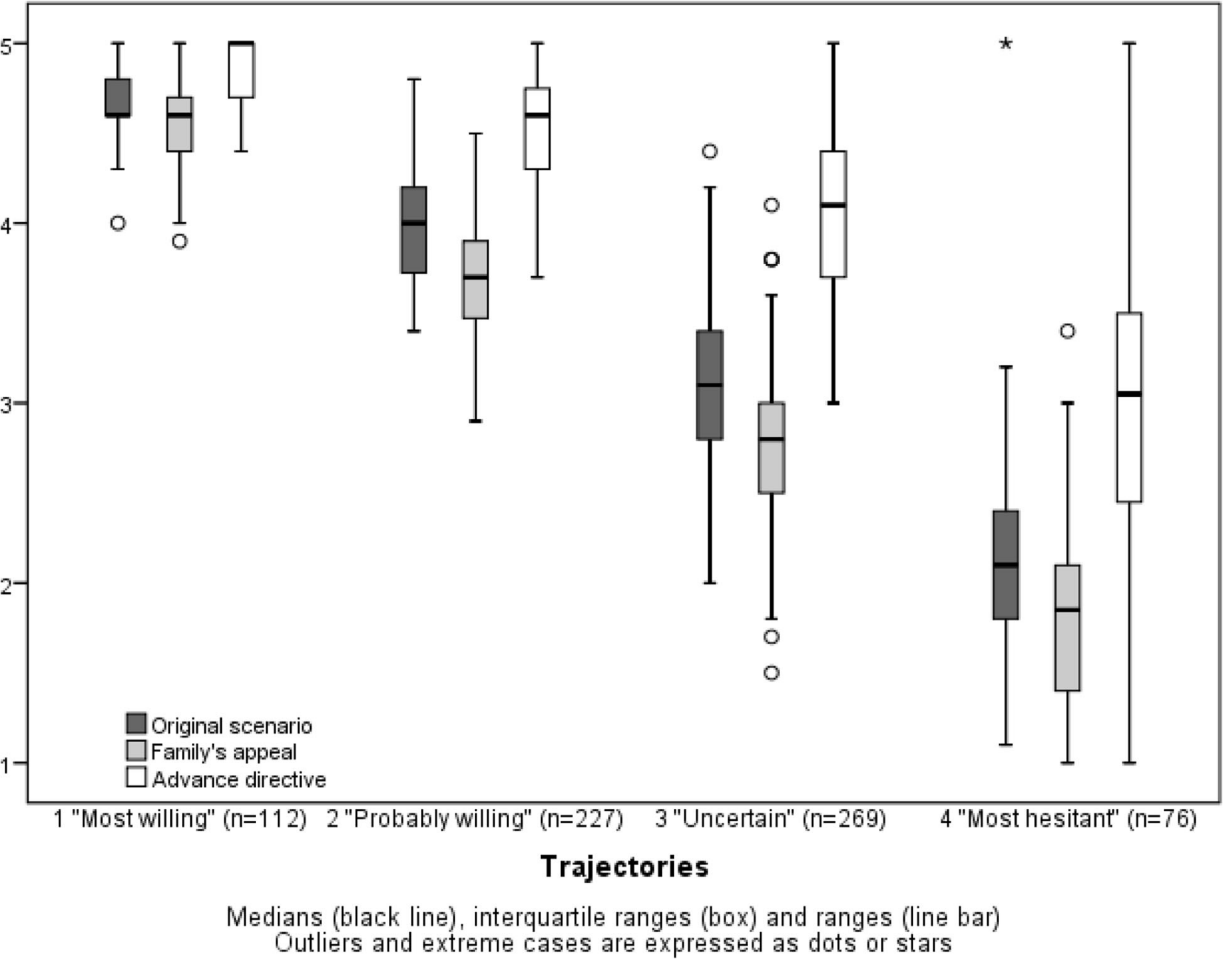
Distribution of the responses (scale from 1 = definitely would not to 5 = definitely would) in the original scenario, family’s appeal and advance directive in the trajectory analysis

When answers were fitted with a trajectory analysis, four differently behaving groups were found (Fig. 2). In the trajectory group 1, responders were consistently willing to withdraw and withhold therapies, and in the trajectory group 2, physicians would probably withdraw and withhold therapies, but their decisions were influenced by the family’s appeal and the advance directive. In contrast, responders encompassed in the trajectory group 3 were either uncertain or chose an aggressive approach in about half of their decisions, and they were more influenced by the advance directive, while physicians in the trajectory group 4 were most hesitant to withdraw and withhold therapies.

Factors associated with the physicians’ willingness to continue or start life-prolonging therapies during EOL-care (belonging to trajectory groups 3 or 4) compared to withhold or withdraw therapies (belonging to trajectory groups 1 or 2) are shown in Table 4. By multivariable analysis, younger age and being an internist, surgeon or GP were independent factors behind the decisions not to withhold - or to withdraw – different interventions. In contrast, gender, being in chief-position, having financial responsibility, or a physician’s own advance directive and experience in EOL-care among relatives did not have independent influence.

**Table 4:** Factors associated with the willingness to continue or start life prolonging therapies (belonging to trajectories 3 or 4) compared to withhold or withdraw therapies (belonging to trajectories 1 or 2) in the patient scenario presented by univariate, age-adjusted and multivariable analysis

		Univariate			Age-adjusted			Multivariate		
	n	OR	(95% CI)	p	OR	(95% CI)	p	OR	(95% CI)	p
Age continuous, years	692	**0.96**	**(0.95–0.98)**	< 0.001						
Age				< 0.001						0.002
25–35	52	**4.71**	**(2.35–9.44)**					**3.19**	**(1.54–6.57)**	
35–49	253	**1.49**	**(1.08–2.05)**					**1.46**	**(1.03–2.06)**	
50–67	387	1.00						1.00		
Sex				0.796			0.433			
Female	425	1.04	(0.77–1.41)		0.88	(0.64–1.21)				
Male	267	1.00			1.00					
Chief-position				0.013			0.208			
No	480	**1.51**	**(1.09–2.11)**		1.25	(0.88–1.76)				
Yes	205	1.00			1.00					
Financial responsibility				0.006			0.083			0.183
No	562	**1.75**	**(1.17–2.62)**		*1.44*	*(0.95–2.19)*		1.35	(0.87–2.08)	
Yes	120	1.00			1.00			1.00		
Own advance directive				0.604			0.932			
No	638	1.17	(0.65–2.09)		1.03	(0.57–1.86)				
Yes	49	1.00			1.00					
End-of-life care among relatives				0.066			0.322			
No	336	*1.32*	*(0.98–1.79)*		1.17	(0.86–1.59)				
Yes	352	1.00			1.00					
Physician group				< 0.001			< 0.001			< 0.001
Competency in PM	66	1.00			1.00			1.00		
Oncologists	92	1.63	(0.78–3.40)		1.39	(0.66–2.93)		1.61	(0.75–3.46)	
Internists	150	**3.92**	**(2.00–7.67)**		**3.85**	**(1.96–7.57)**		**4.27**	**(2.13–8.56)**	
Surgeons	142	**4.53**	**(2.30–8.90)**		**4.37**	**(2.21–8.64)**		**4.51**	**(2.25–9.07)**	
GPs	242	**6.27**	**(3.29–12.0)**		**5.34**	**(2.78–10.3)**		**5.60**	**(2.85–11.0)**	

Significant results (*p* < 0.05) bolded and nearly significant (*p* < 0.10) shown by italic font

Age-adjusted significant (*p* < 0.05) or nearly significant (*p* < 0.10) variables included into the multivariate model. Missing values were not analyzed

PM, Palliative Medicine, GP, General Practitioner

## Discussion

We found that physicians with cPM were more willing to withdraw and withhold life-prolonging therapies, especially intravenous hydration and a nasogastric tube, in a patient scenario representing EOL-care. The family’s request increased the aggressive approach in all physicians, whereas the availability of an advance directive decreased this. Younger age and being an internist, surgeon or GP without cPM were independent factors for responses reflecting willingness to start or continue life-prolonging treatments in multivariable regression analysis.

In this study, the overall willingness to withhold and withdraw therapies in EOL-care was higher in physicians with cPM, measured by mean values of all the answers and in a multivariable regression analysis, although oncologists and cPMs differed only slightly. We used trajectory analysis to take into account all the scenarios in the given order and found a similar pattern across all four groups. Therefore, the groups starting from a low willingness to withhold or withdraw therapies in the original scenario were finally chosen to be presented in the multivariable analysis. In light of previous studies [43–45], it is understandable that physicians with formal training in PM have good ability to consider and communicate the EOL decisions, probably leading to more decisions to withdraw and withhold treatments. We suggest that this willingness is related to the cPM itself as its influence remained also after multivariate analysis taking into account some important background factors in our study. We have to state, however, that we don’t know all the attitudes, which might drive physicians to special education in PM and whether these factors also predispose to withholding and withdrawing life-sustaining treatments.

As our case represented a cancer patient, it is not surprising that responses among physicians with cPM and oncologists were quite similar, although there were differences concerning individual interventions. The relative unwillingness of GPs to make decisions for a palliative approach is a bit concerning, since a vast majority of dying patients in Finland are cared for by GPs. This result was independent of the GPs’ younger age. Our results highlight the need for education in PM starting from medical school and continuing throughout specialty training. In addition, palliative care consultations have shown to be beneficial and they should be offered to all specialities to help complex decision-making in EOL-care [6, 46–48].

Younger age was associated with unwillingness to withhold and withdraw therapies in our study. Age seems to be a contradictory factor in decision-making [49]. In some studies, including our own, older age has been associated with more decisions to withhold or withdraw interventions [50, 51], while in others, younger physicians or trainees make less aggressive decisions [52–54]. Younger physicians have less experience in EOL-care, but on the other hand, PM is currently included in the curriculum of many medical schools, increasing younger colleagues’ awareness of the benefits of palliative care. After 1999 two out of the five medical schools in Finland has included an undergraduate curriculum in PM fulfilling the European recommendations [55, 56]. Our results are in line with other studies showing that gender does not influence the decision-making [51, 54]. Some of the other background factors (such as experience in EOL-care with loved ones or a physician’s own advance directive) did not influence the decision-making in our study, but are not included in previous studies.

Our results imply, that decisions to withhold or withdraw therapies in a clinical practise is mainly driven by medical education and clinical experience of a physician and preferences of a patient rather than doctor’s personal life experience or attitudes.

Advance directive and a healthcare proxy or the family’s opinion have been shown to have marked influence on physicians’ decision-making [12, 31, 35, 57, 58], but there are no earlier studies about this for palliative care physicians. Our study is in line with previous ones [31, 32], since the family’s request for aggressive treatments significantly increased physicians’ willingness to continue or start life-prolonging therapies, and advance directive decreased this. This finding was constant through different physician groups including physicians with cPM. Communication and shared decision-making are very important in EOL-care [20, 49–54, 57, 58], but futile therapies should not be used (even if families have requested them), as stated by the Finnish National Supervisory Authority for Welfare and Health [59]. Therefore, this clear influence of family requests on decision-making is controversial and perhaps an issue needing more attention in the education of PM, which should also introduce legal aspects and official recommendations on decision-making. Knowing a patient’s own will helps in decision-making [31, 32], and an advance directive naturally moves the decisions towards a palliative approach. However, the content of an advance directive presented here did not describe the patient’s will in detail, which is often the case in the real world as well. The understanding of “active treatments” probably influenced the decisions concerning individual therapies in the present study and calls for more detailed advanced care planning and advance directives in clinical practice.

The differences in decision-making between physicians with cPM and others were most striking for nasogastric tube and intravenous hydration. Surgeons, internists and to a lesser extent GPs were unwilling to withdraw hydration, even when an advance directive was found. Artificial nutrition or medically assisted hydration has not been shown to improve survival, quality of life or symptoms in EOL-care, although the evidence about this is scarce [60–64]. There are studies, however, raising concerns about the potential harms, such as increased respiratory secretions, related to hydration during EOL [65]. Although the use of artificial nutrition or intravenous hydration in EOL-care remains controversial, the case scenario in our study represented a dying patient in which these therapies can be considered non-beneficial. The pros and cons of these therapies are included in the formal training in PM, but are probably quite unfamiliar to other physicians.

Supplementary oxygen was the least withdrawn treatment in our study, even among physicians with cPM. This result is in line with reports showing that oxygen is used in more than 70% of patients in EOL-care [66, 67], although the evidence to support this is lacking [68–70]. Perhaps this unwillingness to withdraw oxygen is related to the presumption of its benefit and harmlessness, although it may cause dryness of the mouth and aggravate communication.

In our study antibiotics were withdrawn by about half of the physicians. Use of antibiotics in EOL is controversial, but there is some evidence that antibiotics might relieve symptoms without serious side-effects, which might explain the unwillingness to withdraw them [71, 72]. Internists and GPs were more unwilling to withdraw thrombosis prophylaxis compared to others, probably due to their familiarity with the indications of anticoagulation in the general population. There are no controlled studies to guide when to stop anticoagulation in palliative care, but as our case represented a dying person, withdrawing it can be considered reasonable [73].

The benefits of transfusions in palliative care are experienced briefly and remain controversial [74]. In our study, the physicians with cPM withheld blood transfusions more frequently than others, although the availability of an advance directive increased the willingness to over 90% in all groups.

Pleural drainage can alleviate dyspnoea, but this is an invasive procedure including some risks in EOL-care [75]. Surgeons and GPs were most eager to perform this procedure, which probably reflects their willingness to perform chest X-rays as well. In a Scandinavian study, intensive care physicians withheld laboratory tests [17] more often than all the physicians in our study, which is somewhat surprising. Changing from cure to care might be more complex in a common hospital ward compared to an intensive care unit (ICU), where withdrawing life-supporting treatments commonly leads to relatively rapid patient death.

### Limitations

Some limitations of this study need to be acknowledged. Our response rate (53%) is higher than in many of the recent surveys [31, 37, 76], but still sets a limitation.

Although there might be some nonresponse bias, our responders can be considered a representative sample of Finnish physicians providing insight into their decision-making. The distribution of physician groups in the study equals the distribution of different specialities in Finland [39]. Similarly, the high proportion of female respondents in our study is understandable, since 60% of physicians in Finland are women and female dominance is true among all the specialities studied excluding surgeons [39]. Answers to hypothetical scenarios might differ from physicians’ decision -making in real life situations. In addition, the scenario forced the responder to give simple “yes” or “no” answer without the possibility for example to discuss with the family to achieve shared decision. Further studies on physicians’ decision-making in clinical practice are needed, although this might be difficult to study in large physician groups, as each clinical circumstance is very different. We suggest, however, that the factors behind decision-making remain similar in real life situations and in our hypothetical scenarios. Finally, most of the treatments in our case clearly intend to prolong life (e.g., mechanical ventilation), while some of them may be partly considered as supporting ones (e.g., pleural drainage). Similarly, oxygen or transfusions may be given for symptom relief only or to prolong life, which should be distinguished.

Therefore, “palliative” or “life-prolonging” intent may be questioned in some decisions, but we suggest that the overall tendency to withdraw or withhold therapies in our study reflects reasonable decision-making in EOL-care. The intention itself behind these decisions is an interesting subject for future studies.

## Conclusions

Physicians with special education in palliative medicine are more willing to withdraw and withhold life-prolonging therapies in EOL-care. This is especially true concerning decisions on hydration, artificial nutrition and transfusions. Families’ request and advance directives have a significant influence on decision-making in all physicians.

Younger age and specialty of a physician are main factors influencing the willingness to start or continue life-prolonging treatments. Therefore, education about decision-making in EOL-care should be mandatory at medical schools and in the training of all the specialities facing dying patients. Palliative care consultations might be needed for complex cases of decision -making in EOL-care.

## Additional file

Additional file 1: The parts of the questionnaire reported in this study. (DOCX 23 kb)

## Abbreviations

cPM: Special competency in palliative medicine
EOL: End-of-life
GP: General health care practitioner
ICU: Intensive care unit
IQR: Interquartile range
PM: Palliative medicine

## References

[1] Cardona-Morrell M, Kim J, Turner RM, Anstey M, Mitchell IA, Hillman K (2016). Non-beneficial treatments in hospital at the end of life: a systematic review on extent of the problem. Int J Qual Health Care.

[2] Zimmermann C, Swami N, Krzyzanowska M, Hannon B, Leighl N, Oza A, Moore M, Rydall A, Rodin G, Tannock I, Donner A, Lo C (2014). Early palliative care for patients with advanced cancer: a cluster-randomised controlled trial. Lancet.

[3] Temel JS, Greer JA, Muzikansky A, Gallagher ER, Admane S, Jackson VA, Dahlin CM, Blinderman CD, Jacobsen J, Pirl WF, Billings JA, Lynch TJ (2010). Early palliative care for patients with metastatic non-small-cell lung cancer. N Engl J Med.

[4] Smith TJ, Temin S, Alesi ER, Abernethy AP, Balboni TA, Basch EM, Ferrell BR, Loscalzo M, Meier DE, Paice JA, Peppercorn JM, Somerfield M, Stovall E, Von Roenn JH (2012). American Society of Clinical Oncology provisional clinical opinion: the integration of palliative care into standard oncology care. J Clin Oncol.

[5] Obermeyer Z, Makar M, Abujaber S, Dominici F, Block S, Cutler DM (2014). Association between the Medicare hospice benefit and health care utilization and costs for patients with poor-prognosis cancer. JAMA.

[6] Henson L, Gao W, Higginson I, Smith M, Davies J, Ellis-Smith C, Daveson B (2015). Emergency department attendance by patients with cancer in the last month of life: a systematic review and meta-analysis. Lancet.

[7] Smith S, Brick A, O'Hara S, Normand C (2014). Evidence on the cost and cost-effectiveness of palliative care: a literature review. Palliat Med.

[8] Tan A, Seah A, Chua G, Lim TK, Phua J (2014). Impact of a palliative care initiative on end-of-life care in the general wards: a before-and-after study. Palliat Med.

[9] Daher M (2013). Ethical issues in the geriatric patient with advanced cancer 'living to the end'. Ann Oncol.

[10] Hinkka H, Kosunen E, Metsanoja R, Lammi UK, Kellokumpu-Lehtinen P (2001). To resuscitate or not: a dilemma in terminal cancer care. Resuscitation.

[11] Hinkka H, Kosunen E, Lammi EK, Metsanoja R, Puustelli A, Kellokumpu-Lehtinen P (2002). Decision making in terminal care: a survey of finnish doctors' treatment decisions in end-of-life scenarios involving a terminal cancer and a terminal dementia patient. Palliat Med.

[12] Hinkka H, Kosunen E, Metsanoja R, Lammi UK, Kellokumpu-Lehtinen P (2002). Factors affecting physicians' decisions to forgo life-sustaining treatments in terminal care. J Med Ethics.

[13] Ilemona ER (2014). An appraisal of ethical issues in end-of-life care. Niger J Med.

[14] Parks SM, Winter L (2009). End of life decision-making for cancer patients. Prim Care.

[15] Reichlin M. On the ethics of withholding and withdrawing medical treatment. Multidiscip Respir Med. 2014;9(1):39-6958-9-39. eCollection 2014 10.1186/2049-6958-9-39 PMC4107538 25057360

[16] White B, Willmott L, Cartwright C, Parker MH, Williams G (2014). Doctors’ knowledge of the law on withholding and withdrawing life-sustaining medical treatment. Med J Aust.

[17] Hynninen M, Klepstad P, Petersson J, Skram U, Tallgren M (2008). Process of foregoing life-sustaining treatment: a survey among Scandinavian intensivists. Acta Anaesthesiol Scand.

[18] Solomon MZ, O'Donnell L, Jennings B, Guilfoy V, Wolf SM, Nolan K, Jackson R, Koch-Weser D, Donnelley S (1993). Decisions near the end of life: professional views on life-sustaining treatments. Am J Public Health.

[19] Vincent JL (1999). Forgoing life support in western European intensive care units: the results of an ethical questionnaire. Crit Care Med.

[20] Levin PD, Sprung CL (2005). Withdrawing and withholding life-sustaining therapies are not the same. Crit Care.

[21] Chung GS, Yoon JD, Rasinski KA, Curlin FA (2016). US Physicians' opinions about distinctions between withdrawing and withholding life-sustaining treatment. J Relig Health.

[22] Centeno C, Rodriguez-Nunez A (2015). The contribution of undergraduate palliative care education: does it influence the clinical patient's care?. Curr Opin Support Palliat Care.

[23] Thoonsen B, Vissers K, Verhagen S, Prins J, Bor H, van Weel C, Groot M, Engels Y (2015). Training general practitioners in early identification and anticipatory palliative care planning: a randomized controlled trial. BMC Fam Pract.

[24] Quinn K, Hudson P, Ashby M, Thomas K (2008). "palliative care: the essentials": evaluation of a multidisciplinary education program. J Palliat Med.

[25] Reville B, Reifsnyder J, McGuire DB, Kaiser K, Santana AJ (2013). Education and referral criteria: impact on oncology referrals to palliative care. J Palliat Med.

[26] Hinkka H, Kosunen E, Metsanoja R, Lammi UK, Kellokumpu-Lehtinen P (2002). General practitioners' attitudes and ethical decisions in end-of-life care after a year of interactive internet-based training. J Cancer Educ.

[27] McConigley R, Aoun S, Kristjanson L, Colyer S, Deas K, O'Connor M, Harris R, Currow D, Yates P (2012). Implementation and evaluation of an education program to guide palliative care for people with motor neurone disease. Palliat Med.

[28] Wallace CL (2015). Family communication and decision making at the end of life: a literature review. Palliat Support Care.

[29] Heyland DK, Allan DE, Rocker G, Dodek P, Pichora D, Gafni A (2009). Canadian researchers at the end-of-life network (CARENET): discussing prognosis with patients and their families near the end of life: impact on satisfaction with end-of-life care. Open Med.

[30] Pardon K, Deschepper R, Stichele RV, Bernheim JL, Mortier F, Bossuyt N, Schallier D, Germonpre P, Galdermans D, Van Kerckhoven W, Deliens L (2010). EOLIC-Consortium: preferences of patients with advanced lung cancer regarding the involvement of family and others in medical decision-making. J Palliat Med.

[31] Escher M, Perneger TV, Rudaz S, Dayer P, Perrier A (2014). Impact of advance directives and a health care proxy on doctors' decisions: a randomized trial. J Pain Symptom Manag.

[32] Escher M, Perrier A, Rudaz S, Dayer P, Perneger TV (2015). Doctors’ decisions when faced with contradictory patient advance directives and health care proxy opinion: a randomized vignette-based study. J Pain Symptom Manag.

[33] Shin DW, Cho J, Kim SY, Chung IJ, Kim SS, Yang HK, Ahn E, Park BR, Seo H, Park JH (2015). Discordance among patient preferences, caregiver preferences, and caregiver predictions of patient preferences regarding disclosure of terminal status and end-of-life choices. Psychooncology.

[34] Hong JH, Kwon JH, Kim IK, Ko JH, Kang YJ, Kim HK (2016). Adopting advance directives reinforces patient participation in end-of-life care discussion. Cancer Res Treat.

[35] Horn R (2014). “I don’t need my patients’ opinion to withdraw treatment”: patient preferences at the end-of-life and physician attitudes towards advance directives in England and France. Med Health Care Philos.

[36] Winter L, Parks SM, Diamond JJ (2010). Ask a different question, get a different answer: why living wills are poor guides to care preferences at the end of life. J Palliat Med.

[37] Nakazawa K, Kizawa Y, Maeno T, Takayashiki A, Abe Y, Hamano J, Maeno T (2014). Palliative care physicians’ practices and attitudes regarding advance care planning in palliative care units in Japan: a nationwide survey. Am J Hosp Palliat Care.

[38] Hinkka H, Kosunen E, Lammi UK, Metsanoja R, Kellokumpu-Lehtinen P (2004). Attitudes to terminal patients' unorthodox therapy: Finnish doctors’ responses to a case scenario. Support Care Cancer.

[39] The Finnish Medical Association: Phycisians in Finland, Statistics on phycisians and the health care system 2016. [https://www.laakariliitto.fi/site/assets/files/1268/ll16_tilasto2016_net1_170114.pdf] Accessed 27 Dec 2017.

[40] The Finnish Medical Association: Special education [https://www.laakariliitto.fi/en/medical-education/] Accessed 15 Mar 2016.

[41] Nagin D (2005). Group-based modeling of development.

[42] Leisch F. FlexMix: a general framework for finite mixture models and latent class regression in R. J Stat Soft. 2004;11(8):1–8.

[43] Ahluwalia SC, Tisnado DM, Walling AM, Dy SM, Asch SM, Ettner SL, Kim B, Pantoja P, Schreibeis-Baum HC, Lorenz KA (2015). Association of early patient-physician care planning discussions and end-of-life care intensity in advanced cancer. J Palliat Med.

[44] Brighton LJ, Bristowe K (2016). Communication in palliative care: talking about the end of life, before the end of life. Postgrad Med J.

[45] Chung HO, Oczkowski SJ, Hanvey L, Mbuagbaw L, You JJ (2016). Educational interventions to train healthcare professionals in end-of-life communication: a systematic review and meta-analysis. BMC Med Educ.

[46] May P, Normand C, Morrison RS (2014). Economic impact of hospital inpatient palliative care consultation: review of current evidence and directions for future research. J Palliat Med.

[47] Parikh RB, Kirch RA, Smith TJ, Temel JS (2013). Early specialty palliative care--translating data in oncology into practice. N Engl J Med.

[48] Wachterman MW, Pilver C, Smith D, Ersek M, Lipsitz SR, Keating NL (2016). Quality of end-of-life care provided to patients with different serious illnesses. JAMA Intern Med.

[49] Frost DW, Cook DJ, Heyland DK, Fowler RA (2011). Patient and healthcare professional factors influencing end-of-life decision-making during critical illness: a systematic review. Crit Care Med.

[50] Alemayehu E, Molloy DW, Guyatt GH, Singer J, Penington G, Basile J, Eisemann M, Finucane P, McMurdo ME, Powell C (1991). Variability in physicians’ decisions on caring for chronically ill elderly patients: an international study. CMAJ.

[51] Garland A, Connors AF (2007). Physicians' influence over decisions to forego life support. J Palliat Med.

[52] Larochelle MR, Rodriguez KL, Arnold RM, Barnato AE (2009). Hospital staff attributions of the causes of physician variation in end-of-life treatment intensity. Palliat Med.

[53] Forte DN, Vincent JL, Velasco IT, Park M (2012). Association between education in EOL care and variability in EOL practice: a survey of ICU physicians. Intensive Care Med.

[54] Christakis NA, Asch DA (1995). Physician characteristics associated with decisions to withdraw life support. Am J Public Health.

[55] Elsner F, Centeno C, Cetto G et al: Recommendations of the European Association for Palliative Care (EAPC) For the Development of Undergraduate Curricula in Palliat Med At European Medical Schools. EAPC 2013. [http://www.eapcnet.eu/LinkClick.aspx?fileticket=S1MI-tuIutQ%3d&tabid=1717.] Accessed 02 Jan 2018.

[56] Lehto JT, Hakkarainen K, Kellokumpu-Lehtinen PL, Saarto T (2017). Undergraduate curriculum in palliative medicine at Tampere University increases students’ knowledge. BMC Palliat Care.

[57] Esteve A, Jimenez C, Perez R, Gomez JA (2009). Factors related to withholding life-sustaining treatment in hospitalized elders. J Nutr Health Aging.

[58] Pautex S, Herrmann FR, Zulian GB (2008). Role of advance directives in palliative care units: a prospective study. Palliat Med.

[59] The Finnish National Supervisory Authority for Welfare and Health: Patient’s rights. [http://www.valvira.fi/web/en/healthcare/patient_rights] Accessed 09 May 2017.

[60] Finucane TE, Christmas C, Travis K (1999). Tube feeding in patients with advanced dementia: a review of the evidence. JAMA.

[61] Good P, Cavenagh J, Mather M, Ravenscroft P (2008). Medically assisted hydration for palliative care patients. Cochrane Database Syst Rev.

[62] Bruera E, Hui D, Dalal S, Torres-Vigil I, Trumble J, Roosth J, Krauter S, Strickland C, Unger K, Palmer JL, Allo J, Frisbee-Hume S, Tarleton K (2013). Parenteral hydration in patients with advanced cancer: a multicenter, double-blind, placebo-controlled randomized trial. J Clin Oncol.

[63] Goldberg LS, Altman KW (2014). The role of gastrostomy tube placement in advanced dementia with dysphagia: a critical review. Clin Interv Aging.

[64] Good P, Richard R, Syrmis W, Jenkins-Marsh S, Stephens J (2014). Medically assisted nutrition for adult palliative care patients. Cochrane Database Syst Rev.

[65] Fritzson A, Tavelin B, Axelsson B (2015). Association between parenteral fluids and symptoms in hospital end-of-life care: an observational study of 280 patients. BMJ Support Palliat Care.

[66] Campos-Calderon C, Montoya-Juarez R, Hueso-Montoro C, Hernandez-Lopez E, Ojeda-Virto F, Garcia-Caro MP (2016). Interventions and decision-making at the end of life: the effect of establishing the terminal illness situation. BMC Palliat Care.

[67] Sato K, Miyashita M, Morita T, Tsuneto S, Shima Y (2016). End-of-life medical treatments in the last two weeks of life in palliative care units in Japan, 2005-2006: a Nationwide retrospective cohort survey. J Palliat Med.

[68] Abernethy AP, McDonald CF, Frith PA, Clark K, Herndon JE, Marcello J, Young IH, Bull J, Wilcock A, Booth S, Wheeler JL, Tulsky JA, Crockett AJ, Currow DC (2010). Effect of palliative oxygen versus room air in relief of breathlessness in patients with refractory dyspnoea: a double-blind, randomised controlled trial. Lancet.

[69] Campbell ML, Yarandi H, Dove-Medows E (2013). Oxygen is nonbeneficial for most patients who are near death. J Pain Symptom Manag.

[70] Uronis HE, Currow DC, McCrory DC, Samsa GP, Abernethy AP (2008). Oxygen for relief of dyspnoea in mildly- or non-hypoxaemic patients with cancer: a systematic review and meta-analysis. Br J Cancer.

[71] Helde-Frankling M, Bergqvist J, Bergman P, Bjorkhem-Bergman L. Antibiotic treatment in end-of-life Cancer patients-a retrospective observational study at a palliative Care Center in Sweden. Cancers (Basel). 2016;8(9) 10.3390/cancers8090084. 10.3390/cancers8090084 PMC5040986 27608043

[72] Rosenberg JH, Albrecht JS, Fromme EK, Noble BN, McGregor JC, Comer AC, Furuno JP (2013). Antimicrobial use for symptom management in patients receiving hospice and palliative care: a systematic review. J Palliat Med.

[73] O'Brien CP (2011). Withdrawing medication: managing medical comorbidities near the end of life. Can Fam Physician.

[74] Uceda Torres ME, Rodriguez Rodriguez JN, Sanchez Ramos JL, Alvarado Gomez F (2014). Transfusion in palliative cancer patients: a review of the literature. J Palliat Med.

[75] Strasser F, Blum D, Bueche D (2010). Invasive palliative interventions: when are they worth it and when are they not?. Cancer J.

[76] Cartwright CM, White BP, Willmott L, Williams G, Parker MH (2016). Palliative care and other physicians' knowledge, attitudes and practice relating to the law on withholding/withdrawing life-sustaining treatment: survey results. Palliat Med.

