# Supplementary material for: Does special education in palliative medicine make a difference in end-of-life decision-making?
Source: BMC Palliat Care. 2018 Jul 18;17:94. doi: 10.1186/s12904-018-0349-6 (PMC6052558; doi:10.1186/s12904-018-0349-6)
Supplement: Supplementary file 1 — The parts of the questionnaire reported in this study. (DOCX 23 kb) [file 12904_2018_349_MOESM1_ESM.docx]

**Additional file 1**

**The parts of the questionnaire reported in this study**

Answer the questions based on the following patient scenario in the given order. Circle the right option. Do not change your answer once decided.

**Scenario**: A 62-year-old male patient with pulmonary cancer and metastases. He is admitted to hospital ward and is receiving high-dose morphine medication. Due to respiratory weakening he became comatose last night. He also suffers from severe anemia and has abundant pleural effusion and fever. After the presentation there is a question about treatment decision: Which of the following treatments already started (*) or planned would you withhold or withdraw? There is no possibility to discuss the matter with the family and there is no advance directive.

Would you withhold or withdraw any of the following treatments; express your decision on the scale 1-5.

|  |  | I definitely would not withhold or withdraw* |  |  |  | I definitely would withhold or withdraw* |
| --- | --- | --- | --- | --- | --- | --- |
| a) | antibiotics (*) | 1 | 2 | 3 | 4 | 5 |
| b) | mechanical ventilation (*) | 1 | 2 | 3 | 4 | 5 |
| c) | blood transfusion | 1 | 2 | 3 | 4 | 5 |
| d) | pleural drainage | 1 | 2 | 3 | 4 | 5 |
| e) | chest x-ray examination | 1 | 2 | 3 | 4 | 5 |
| f) | laboratory tests | 1 | 2 | 3 | 4 | 5 |
| g) | IV hydration (*) | 1 | 2 | 3 | 4 | 5 |
| h) | nasogastric tube (*) | 1 | 2 | 3 | 4 | 5 |
| i) | thrombosis prophylaxis (*) | 1 | 2 | 3 | 4 | 5 |
| j) | supplementary oxygen (*) | 1 | 2 | 3 | 4 | 5 |

1. The patient’s daughters come to you distressed and crying, expressing their hope that everything possible will be done to save their fathers life.

Would you withhold or withdraw any of the following treatments; express your decision on the scale 1-5.

|  |  | I definitely would not withhold or withdraw* |  |  |  | I definitely would withhold or withdraw* |
| --- | --- | --- | --- | --- | --- | --- |
| a) | antibiotics (*) | 1 | 2 | 3 | 4 | 5 |
| b) | mechanical ventilation (*) | 1 | 2 | 3 | 4 | 5 |
| c) | blood transfusion | 1 | 2 | 3 | 4 | 5 |
| d) | pleural drainage | 1 | 2 | 3 | 4 | 5 |
| e) | chest x-ray examination | 1 | 2 | 3 | 4 | 5 |
| f) | laboratory tests | 1 | 2 | 3 | 4 | 5 |
| g) | IV hydration (*) | 1 | 2 | 3 | 4 | 5 |
| h) | nasogastric tube (*) | 1 | 2 | 3 | 4 | 5 |
| i) | thrombosis prophylaxis (*) | 1 | 2 | 3 | 4 | 5 |
| j) | supplementary oxygen (*) | 1 | 2 | 3 | 4 | 5 |

1. There is a written advance directive in the patient’s medical chart in which he expresses his wish that all active treatment should be withdrawn, if there is no hope of recovery.

Would you withhold or withdraw any of the following treatments; express your decision on the scale 1-5.

|  |  | I definitely would not withhold or withdraw* |  |  |  | I definitely would withhold or withdraw* |
| --- | --- | --- | --- | --- | --- | --- |
| a) | antibiotics (*) | 1 | 2 | 3 | 4 | 5 |
| b) | mechanical ventilation (*) | 1 | 2 | 3 | 4 | 5 |
| c) | blood transfusion | 1 | 2 | 3 | 4 | 5 |
| d) | pleural drainage | 1 | 2 | 3 | 4 | 5 |
| e) | chest x-ray examination | 1 | 2 | 3 | 4 | 5 |
| f) | laboratory tests | 1 | 2 | 3 | 4 | 5 |
| g) | IV hydration (*) | 1 | 2 | 3 | 4 | 5 |
| h) | nasogastric tube (*) | 1 | 2 | 3 | 4 | 5 |
| i) | thrombosis prophylaxis (*) | 1 | 2 | 3 | 4 | 5 |
| j) | supplementary oxygen (*) | 1 | 2 | 3 | 4 | 5 |

Next there are questions about your attitudes. Draw a vertical line to the scale on a place that best describes your attitude.

1. Active euthanasia is reprehensible

Definitely agree Definitely disagree

I------------------------------------------------------------------------------------I

1. Withdrawal of life-sustaining treatments is reprehensible

Definitely agree Definitely disagree

I------------------------------------------------------------------------------------I

1. Assisted suicide is reprehensible

Definitely agree Definitely disagree

I------------------------------------------------------------------------------------I

1. End-of-life care is satisfying

Definitely agree Definitely disagree

I------------------------------------------------------------------------------------I

1. People should pay costs of factitious diseases by themselves

Definitely agree Definitely disagree

I------------------------------------------------------------------------------------I

1. Advance directives have been helpful in my decisions

Definitely agree Definitely disagree

I------------------------------------------------------------------------------------I

1. Good palliative care enables good death

Definitely agree Definitely disagree

I------------------------------------------------------------------------------------I

1. Physicians can’t estimate cancer pain

Definitely agree Definitely disagree

I------------------------------------------------------------------------------------I

1. Religion has influence when I make ethical decisions

Definitely agree Definitely disagree

I------------------------------------------------------------------------------------I

1. Being a doctor gives me satisfaction

Definitely agree Definitely disagree

I------------------------------------------------------------------------------------I

1. My health is excellent

Definitely agree Definitely disagree

I------------------------------------------------------------------------------------I

1. I feel burn out, tired to work

Definitely agree Definitely disagree

I------------------------------------------------------------------------------------I

1. I’m pleased with my salary

Definitely agree Definitely disagree

I------------------------------------------------------------------------------------I

1. It is waste of resources to treat patients > 80 years in ICU

Definitely agree Definitely disagree

I------------------------------------------------------------------------------------I

Answer to the next questions as asked. When options given, please circle the right option.

Age:_____________ Gender: female male Year of graduation:______________________

Amount of administrative work _______________ hours per week

Main working place: Outpatient unit Hospital Other:_______________________

Do you have your own advance directive? yes no

Have you had professional supervision? yes no

Are you in a chief position in your main job? yes no

Are you responsible of a budget? yes no

Have you taken care of end-of-life (terminal) patients

during the last two years? yes no

Do you have experience of end-of-life (terminal)

care among close friends or family members? yes no

Finally some life values. Choose an option that is closest to your thinking.

|  | Not at all important | Not very important | Quite important | Very important |
| --- | --- | --- | --- | --- |
| Length of life | 1 | 2 | 3 | 4 |
| Health | 1 | 2 | 3 | 4 |
| Family | 1 | 2 | 3 | 4 |
| Clean environment | 1 | 2 | 3 | 4 |
| High standard of living | 1 | 2 | 3 | 4 |
| Faith in God | 1 | 2 | 3 | 4 |
| Success in professional career | 1 | 2 | 3 | 4 |
